# Viral and Clinical Oncology of Head and Neck Cancers

**DOI:** 10.1007/s11912-022-01263-7

**Published:** 2022-03-26

**Authors:** Peter Goon, Matthias Schürmann, Felix Oppel, SenYao Shao, Simon Schleyer, Christoph J. Pfeiffer, Ingo Todt, Frank Brasch, Lars-Uwe Scholtz, Martin Göerner, Holger Sudhoff

**Affiliations:** 1 University Hospital Department of Otolaryngology & Head and Neck Surgery, Campus Klinikum Bielefeld Mitte, University Hospital OWL, University of Bielefeld, Teutoburger Str. 50, 33604 Bielefeld, Germany; 2 Centre for Pathology and Molecular Oncology, Campus Klinikum Bielefeld, University Hospital OWL, Teutoburger Str. 50, 33604 Bielefeld, Germany; 3 Department of Clinical Oncology, Campus Klinikum Bielefeld, University Hospital OWL, Teutoburger Str. 50, 33604 Bielefeld, Germany

**Keywords:** Head and neck cancers, EBV, HPV

## Abstract

**Purpose of Review:**

This study assesses the current state of knowledge of head and neck squamous cell carcinomas (HNSCC), which are malignancies arising from the orifices and adjacent mucosae of the aerodigestive tracts. These contiguous anatomical areas are unique in that 2 important human oncoviruses, Epstein-Barr virus (EBV) and human papillomavirus (HPV), are causally associated with nasopharyngeal and oropharyngeal cancers, respectively. Mortality rates have remained high over the last 4 decades, and insufficient attention paid to the unique viral and clinical oncology of the different subgroups of HNSCC.

**Recent Findings:**

We have compared and contrasted the 2 double-stranded DNA viruses and the relevant molecular oncogenesis of their respective cancers against other head and neck cancers. Tobacco and alcohol ingestion are also reviewed, as regard the genetic progression/mutation accumulation model of carcinogenesis. The importance of stringent stratification when searching for cancer mutations and biomarkers is discussed. Evidence is presented for a dysplastic/pre-invasive cancerous phase for HPV+ oropharyngeal cancers, and analogous with other HPV+ cancers. This raises the possibility of strategies for cancer screening as early diagnosis will undoubtedly save lives.

**Summary:**

Staging and prognostication have changed to take into account the distinct biological and prognostic pathways for viral+ and viral− cancers. Diagnosis of pre-cancers and early stage cancers will reduce mortality rates. Multi-modal treatment options for HNSCC are reviewed, especially recent developments with immunotherapies and precision medicine strategies. Knowledge integration of the viral and molecular oncogenic pathways with sound planning, hypothesis generation, and clinical trials will continue to provide therapeutic options in the future.

## Introduction

Head and neck squamous cell carcinoma (HNSCC) is the sixth most common type of malignant tumour in the world, with 890,000 new cases and 460,000 deaths attributed to this cancer in 2018 [1, 2]. The incidence of HNSCC has risen steadily over the last few decades with almost all regions of the world reporting increases, and is predicted to increase by up to 30% from current levels to approximately 1.08 × 10^6^ new cases per annum by 2030 (GLOBOCAN — Global Cancer Observatory [3]). HNSCCs encompass the SCCs developing from the epithelium of the oral cavity, nasopharynx, oropharynx, hypopharynx, and larynx [4••].

The major risk factors known for this group of tumours are varied and disparate, and they include intermittent and frequent exposure to carcinogens via oral ingestion of alcohol, tobacco smoking/chewing/eating, marijuana smoking/eating (highly likely to be carcinogenic as cannabis smoking contains tar plus many of the known carcinogens found in tobacco smoke), smoking/eating or chewing Betel (*Piper betle*) leaves/Areca (*Areca catechu*) nuts/slaked lime +/− tobacco +/− other spices or herbs in different local customised mixtures [5].

Other important risk factors (which are increasingly recognised) include air pollution with chemicals and particulate matter especially noted in some of the world’s megacities, ageing (although this may just signify longer exposure to the aforementioned carcinogens in combination with decreased immune surveillance as the immune system ages), poor oral hygiene and dental care (including the oral and pharyngeal microbiome/virome), chronic inflammation including reflux oesophagitis, and poor dietary intake.

Oncogenic human viruses have been identified and increasingly recognised since the discovery of the first, when Epstein-Barr virus was observed within Burkitt Lymphoma cells in 1964 using electron microscopy by Anthony Epstein, Bert Achong, and Yvonne Barr [6]. Since that seminal work, there have been extensive searches for viruses associated with human cancers (since the initial discovery of Rous Sarcoma virus in chickens by Peyton Rous in 1911), and there are now 8 recognised human oncoviruses (5 DNA (hepatitis B virus (HBV), human papillomavirus (HPV), Epstein-Barr virus (EBV), Kaposi’s sarcoma herpes virus (KSHV or human herpes virus 8), Merkel cell polyomavirus (MCPV), and 3 RNA viruses (hepatitis C virus (HCV) and human T-lymphotropic virus types 1 and 2 (HTLV-1 and HTLV-2)) which have strong evidence for their causality of different cancers. Intensive research into viral oncology has been ongoing, and there will be undoubtedly more discoveries in this exciting and dynamic field. It was estimated that approximately 10.2% of cancers worldwide are virally driven in 2012 [7], and if we assume at least this frequency for 2020 (the overall trend for these carcinogenic virus-induced cancers was increasing over time), this should equate to over 1,968,600 cases annually [1, 3].

Host genetic factors also have been shown to be of great importance. This is illustrated best by Fanconi anaemia (FA), one of the best studied inherited cancer-prone syndromes [8]. The overall risk of a Fanconi anaemia patient developing an HNSCC (2/3 of cases are oral cavity tumours (most at the tongue margins and gingival areas) has been estimated at *>700× higher* than the background population risk of an age, gender, and birth-matched cohort in North America [9]. FA patients also have an increased risks of developing other cancers such as AML (scute myeloid leukaemia) (>700×), oesophagus (>2000×), vulvar (>4000×), and liver cancers (>300×). There are other inherited syndromes with inherent genetic instability, and these also demonstrate an increased susceptibility to developing early malignancies; these include Bloom syndrome, Werner syndrome, and Ataxia Telangiectasia.

## Pathogeneses of HNSCC

The temporal pathway of mutation accumulation (genetic progression model) for clonal cancer development is currently the main hypothesis thought to be important for HNSCC. The cancer cell of origin is dependent on the anatomical site and the epithelium involved, and the triggers for development of neoplasia are the environmental and viral carcinogens. Normal stem cells or progenitor cells at each site sustain and develop mutations that allow progression along the temporal mutation pathway, and give rise to the cancer stem cell.

**Fig. 1:**
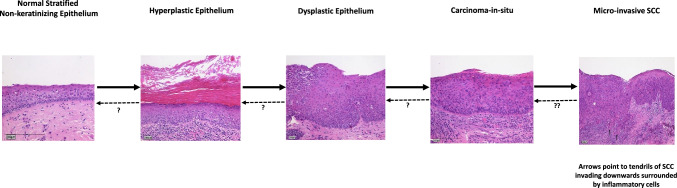
Mutation accumulation/genetic progression model of HNSCC development

There are strong histological and genetic evidence for this model. Califano et al. [9] demonstrated that there significant mutations which were enriched at each histological stage, developing from a normal cell to hyperplasia, to dysplasia, to carcinoma in situ, and then invasive carcinoma (see Figure 1) They investigated 10 major chromosomal loci (which had been documented previously) for allelic loss using microsatellite analyses and more complete lists to date have been published [4, 10].

There are some evidence for a neoplastic stage resolving spontaneously (spontaneous regression), most commonly from the early stage cervical neoplasia model (CIN 1–2 back to normal) and much less common for invasive disease, although case reports have been documented and collated in the literature [11–13].

The term “field cancerisation” was first coined by Slaughter, Southwick, and Smejkal in 1953 [14] after they discovered that large areas of grossly normal looking (to the naked eye) epithelium around the excised tumours were almost always microscopically abnormal, indicating that large surface areas of tissue had undergone carcinogenic exposure, while the main tumour body (or tumours) was testament to the development of malignancy from within the damaged areas. This concept has been largely accepted in the field, and used to explain second primary tumours (SPTs) where synchronous and/or metachronous tumours have arisen, or for recurrent tumour (post-treatment) growth.

## Viruses as Carcinogens

The International Agency for Research on Cancer (IARC) classified Epstein-Barr virus (EBV) (originally labelled human herpes virus 4) and human papillomavirus (HPV) as group 1 carcinogens (agents that cause cancer) in 1997, and 1995 respectively. These 2 viral carcinogens have been identified as important causes of cancers in humans since their discoveries, and huge progress and knowledge have accumulated in the delineation of the molecular pathways of the oncogenic process. Uniquely, these two double-stranded DNA viruses have been strongly and causatively associated to tumours of the head and neck region, over the last few decades.

In Epstein’s own account [15], the serendipitous discovery of EBV was made possible after prolonged culture and transportation conditions (agitation) when the plane carrying the Burkitt Lymphoma biopsies from Uganda was diverted by fog from London to Manchester. After finally arriving in London’s Middlesex hospital, the cloudy fluid was thought to be contaminated with bacterial infection and therefore useless, but he was astounded when he found large numbers of free-floating lymphoma cells in suspension. Suspension culture became the norm for culture of lymphocytic cell lines from then onwards. Herpes-virus like particles were first seen on 24 February 1964, and the discovery reported on 28 March 1964 [6].

It has been known for a long time epidemiologically that the aetiology of cervical carcinoma was most likely via a sexually transmitted agent, since it was almost never found in women who professed to be virgins or in nuns [16]. For years, human herpes virus 2 ( also known as herpes simplex virus type 2) was strongly thought to be agent responsible [17], but the evidence was always incomplete, unconvincing and controversial (Robert Koch’s postulates for causality were not fulfilled). Finally, in 1983, Harald zur Hausen’s team [18] published that they had succeeded in finding HPV DNA in a large number of cervical carcinomas by using a DNA probe from HPV 11 (low-risk HPV type) under non-stringent hybridisation conditions. This HPV type was subsequently designated HPV 16, and found to be hybridising under stringent conditions to cervical cancers (Germany, Kenya & Brazil (11/18), in situ cervical carcinoma (2/9), cervical dysplasia (2/20), vulvar cancer (2/7), penile cancer (1/4), and genital warts (2/33)[18]l. HPV 16 is now known to be causally associated with >50% of all cervical carcinomas (essentially all cervical cancers are HPV+) and >90% of HPV+ HNSCC.

## Structures

See Fig. 2.

**Fig. 2:**
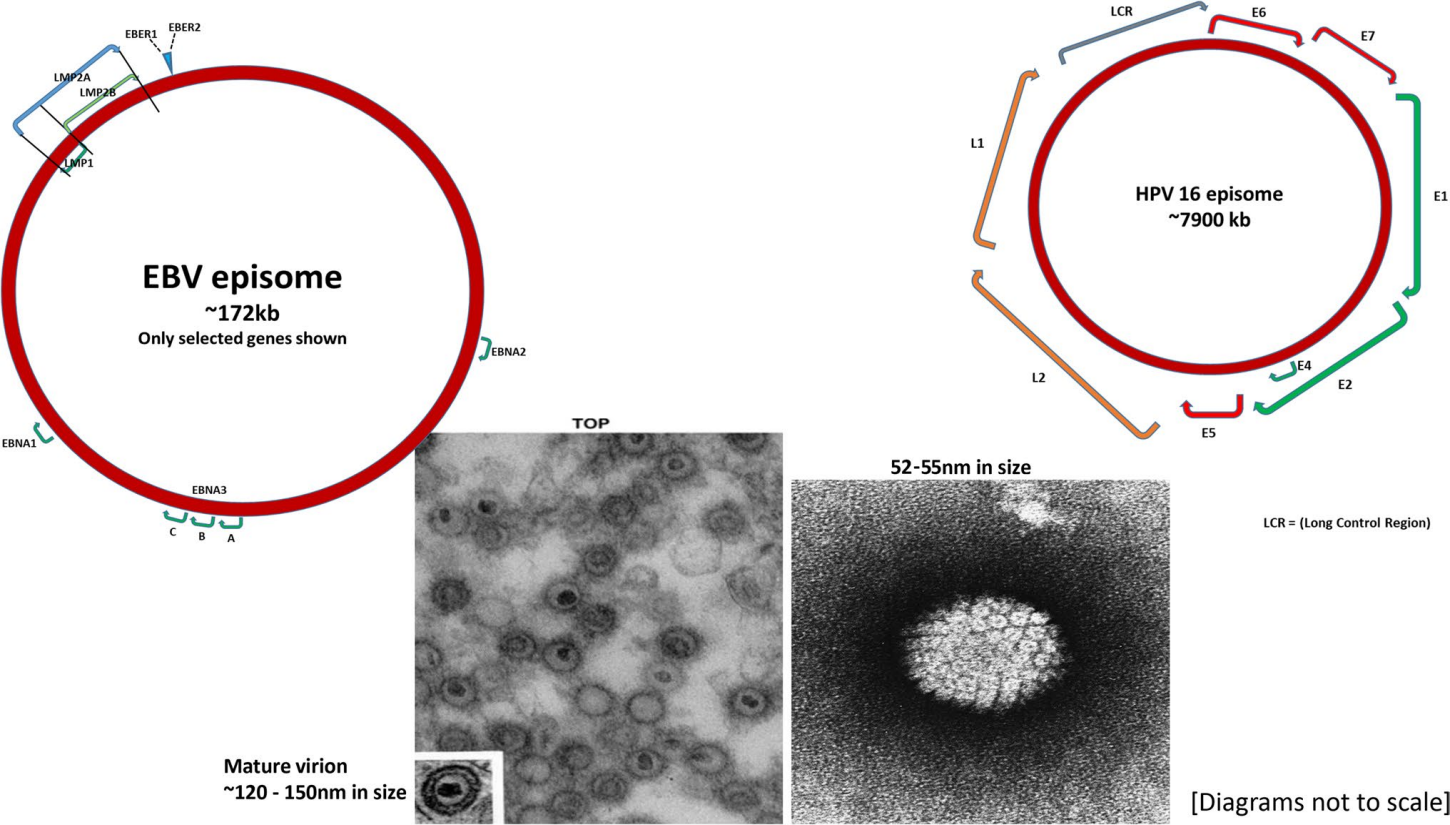
Genetic and electron microscopic structures of EBV and HPV

## Comparison of EBV+/− and HPV+/− HNSCC

See Table 1.

**Table 1:** Comparison of certain features in viral +/− head and neck cancers

	EBV+ NPC	EBV− NPC	HPV+ HNSCC (most oropharyngeal)	HPV− HNSCC
Trigger	DS-DNA virus	Sporadic, likely tobacco/alcohol/other carcinogens	DS-DNA virus	Tobacco/alcohol/other carcinogens
WHO carcinogen group	Group 1 carcinogen	Group 1 carcinogens	Group 1 carcinogen	Group 1 carcinogens
Virus genome size	Size ~172 kb	NA	Size ~8 kb	NA
Virus location in cell	Episomal genome	NA	Mostly integrated into cellular genome (some episomal)	NA
Differentiation	Non-keratinising or undifferentiated SCC	Moderate to well differentiated SCC	Basaloid or undifferentiated SCC	Mostly moderate to well-differentiated SCC
Age at diagnosis	~ 50 years	~ >65 years	~ 53 years	~ > 65 years
Endemicity	High incidence rates in Southern China, SouthEast Asia, moderate rates in North Africa (Maghreb)	>100 fold lower in non-endemic areas	30–70% of annual oropharyngeal SCCs in Northern, Western Europe, North America	Dominant fraction of non-oropharyngeal cancers, and other head and neck anatomical sites
Sensitivity to treatment	Sensitive to radiochemotherapy (wtTP53)	Less sensitive	Sensitive to radiochemotherapy (wtTP53)	Less sensitive
Gender ratio	Male preponderance	Male preponderance	Male preponderance	Male preponderance
Targeted therapy potential	Viral (foreign) antigens and some endogenous mutations may be suitable as targets for screening or therapy	NA	Viral (foreign) antigens suitable as targets for screening or therapy	NA
Vaccine	Prophylactic vaccine not available (NA)	NA	Prophylactic vaccine available	NA

## EBV+/− Nasopharyngeal Carcinoma

### Epidemiology

This cancer is endemic amongst the populations of Southern China and Southeast Asia (especially the Cantonese populations in these regions where the male ASR (age-standardised incidence rates) is estimated at ~13–25/10^5^) in 2012) and in North Africa (Maghreb) (male ASR of ~4–6/10^5^) [19]. In the West (Europe, North America, Australia-New Zealand), this subtype of HNSCC is rarely found amongst those populations and only then, usually in patients from immigrant communities. Almost all (>95%) of cases from endemic regions are EBV+ non-keratinising (type 2) or poorly differentiated carcinomas (type 3), while cases in non-endemic areas are typically EBV− keratinising carcinomas with well-moderately differentiated SCC histology (type 1).

Research has demonstrated that descendants of emigrants from EBV+ NPC endemic regions persist in having a higher incidence of this type of cancer despite settling in a non-endemic region, and that risk decreases by the generation. This suggests significant genetic factors involved but also that environmental factors play important roles [20, 21].

The discovery of familial clusters in Southern China demonstrated that having a first degree relative with NPC would attribute a lifetime risk of >5% to an individual of developing NPC. Odds ratios ranged from 2 to 20 [22, 23]. HLA immunophenotyping bear these observations out. HLA-A2-B46 and B17 are associated with 2–3 fold increase in risk for NPC in the Chinese and other high-risk Asian populations, whereas HLA-B5 in Caucasians also carries an increased risk. HLA-A11 in all races, B13 in the Chinese and Tunisians, and A2 in non-Chinese all carry a reduced risk of NPC [24].

A strong contribution towards the development of NPC by environmental factors has been shown by association studies for the consumption (especially in the young) of salted fish, salted meat and vegetables, soya sauce, harissa and quaddid (spiced meat preserved in olive oil), other spice mixtures for stews from the Maghreb, and other dietary agents [25, 26]. Tobacco smoking is a definite contributory risk factor, with an increased risk of 2–6 fold for NPC [27]. Occupational exposure to wood/wood-dust/iron filings is also thought to be risk factors [28, 29].

The mortality rates (5 year survival rates) are approximately 50–60% for EBV+ NPC due to late diagnoses as most cancers are diagnosed at Stages III and IV. This is due to the generally asymptomatic but highly malignant nature of this tumour. EBV+ NPC patients also tend to be much younger than the sporadic non-endemic EBV− NPC in the West (> 65 years), and the median age at diagnosis is 50 years (45–55 years) in males (males 2–3x : 1 female)[4, 30].

### Viral Oncology

EBV is unmatched for causing multiple different cancers and lymphoproliferative disorders in different anatomical sites, and in different immune states of humans. Indeed, no single virus or infectious agent can account for so many different disorders, i.e., infectious mononucleosis (50% of 1° infections are asymptomatic, and especially in younger patients. The vigorous immune response towards the virus in teenagers and young adults appears to cause classic “Mono” syndrome), B cell lymphoproliferative disorders include Burkitt’s lymphoma, Hodgkin’s lymphoma, and post-transplant lymphoproliferative disorder (PTLD). T and NK cell lymphoproliferative disorders have also been reported, and these include cutaneous T cell lymphoproliferative disorder, peripheral T cell lymphoma, aggressive NK cell leukaemia/lymphoma, angioimmunoblastic T cell lymphoma, and extranodal nasal type NK/T cell lymphoma. Epithelial malignancies shown to be associated with EBV include gastric carcinoma and nasopharyngeal carcinoma [31, 32].

EBV oncogenesis in NPC has largely been imputed from work done in B cell cancers, and the B cell is the main cell of tropism (CD21 (dominant receptor), CD35, and HLAII are the cell receptors to which EBV binds and enters the B cell), while EBV infection of epithelial cells appears to be much less efficient [33, 34]. In vitro studies demonstrate that cell-free virion infection of epithelial cells is much less efficient than cell-cell contact between EBV producing cell lines and primary or epithelial cell lines. The cell receptor on epithelial cells has recently been identified as Ephrin receptor A2 (EphA2) [35•], and this seminal work confirmed that EBV glycoproteins gH/gL and gB directly attach to EphA2 on both gastric and nasopharyngeal epithelial cells, and this allows binding and subsequent internalisation. B cell and T cell entry by EBV has been characterised, and EBV gp 350/gp 220 allows binding to the lymphocyte cell surface CD21 and/or CD35, while gp42 binds to HLA II for fusion and internalisation [36, 37•].

The generation of neutralising antibodies to these EBV glycoproteins via vaccine development and immunisation is a huge target of translational medical research, as it would allow prevention of EBV infection and its subsequent sequelae and cancers.

Establishment of latent infection once EBV has entered the cell is a prerequisite for long-term infection as the default infection programme in normal pharyngeal epithelial cells is lytic. This is in contrast to the cancer cells found in epithelial cancers where the infection is latent, and therefore allows for long-term survival of the cell (no lysis). The actual mechanism and control of switching from lytic to latent infection are unclear. There are 3 types of latency programmes known at present: type 1 is the classic Burkitt’s Lymphoma from B cells, and only EBERs (Epstein-Barr virus–encoded small RNAs) and EBNA1 (EBV nuclear antigen 1) are expressed. These 2 are expressed in all 3 latency programmes. Type 2 latency is seen in epithelial cancers, and includes both gastric carcinoma and NPC; LMP1 (Latent membrane protein 1) and LMP2A, LMP2B, BARTs (*BamHI A region rightward transcripts*), and BART miRNAs are also expressed. Type 3 latency is seen in the immunocompromised lymphomas (primary cerebral lymphoma, etc) including HIV and iatrogenically induced immunosuppression such as transplant patients.

Whole exome sequencing (WES) and whole genome sequencing (WGS) are being increasingly utilised to characterise the mutations driving EBV+ NPC. No large published studies on EBV− NPC are available. It is inevitable that the oncogenic pathways leading to histological type 1 keratinising EBV− NPC, and that type 2 differentiated and type 3 undifferentiated EBV+ NPCs will be different.

These EBV+ NPCs are sensitive to radiotherapy and chemotherapy, and this may be partially explained by the fact that wild type *wtTP53* is found in >90% of these tumours [38], and its key role in apoptosis is still functional. Nutlin-3 is a small molecule inhibitor (targets p53-MDM2 interaction) [39] which has been shown to potentiate the p53 apoptosis pathway and sensitises the tumour cells to cisplatin cytotoxicity in metastatic and recurrent tumours [40, 41]. The mutational burden found in EBV+ NPC also appears to be relatively decreased compared to other malignancies [42] suggesting that the viral oncogenic pathway predominantly causes this rapidly replicating and malignant phenotype.

Available molecular evidence is categorised here under the banner of EBV+/− NPC, but it is likely that further sub-categorisation under smoking, alcohol, and other environmental triggers would be relevant to understand the specific oncogenic pathways involved. The most prominent EBV oncogenic drivers so far detected in NPC via WGS appear to be latent membrane protein 1(LMP1) oncogene and Epstein-Barr virus–encoded small RNAs (*EBERS*). There is significant overexpression of *LMP1* (which drives the NF-κB pathway most strongly), and these studies [38, 42] have found that there was mutual exclusion of NF-κB pathway genomic aberrations when LMP1 overexpression was detected. The authors concluded that 70% of their 111 cases were either LMP1 over-expressed or contained significant NF-κB pathway negative regulator mutations (loss-of-function), suggesting that the NF-κB pathway activation is strongly implicated in driving a majority of EBV+ NPC. Negative regulators of this pathway demonstrating numerous loss-of-function mutations included *CYLD* (18.6%), *TRAF3* (17.5%), *NFKBIA* (6.7%), and *NLRC5* (4.8%).

*EBNA1* as an EBV oncogenic driver is also involved, since it is known to be expressed in all EBV-associated cancers. Other latency type 2 EBV proteins or nucleotides that would be involved in driving EBV+ NPC would be *LMP2A* and *LMP2B* and *BamHI A region rightward transcripts* (BARTs) and *BART miRNAs*. All of these oncogenic drivers have had numerous interactions and pathways delineated, but almost certainly, many more actions remain to be found. EBV encodes for >85 genes, and it is clear that EBV viral genetics has evolved and selected for its huge success in infecting >90% of humans by the time they reach adulthood. Much research still needs to be done to clarify the functions of the numerous EBV genes involved in NPC pathogenesis.

*EBERS* also appear to increase NF-κB pathway activation through their interactions with *TLR3* in EBV+ NPC, and this appears to be via a positive feedback loop with *LMP1* [43]. However, other major signalling pathways such PI3K (20.7%), MAPK (11.7%), JAK/STAT (10.8%), NOTCH (10.8%), and WNT (10.8%) were also shown to carry significant mutations, demonstrating the heterogeneity of the oncogenic pathways involved. MHC Class I mutations were also found in 28.8% of patients, demonstrating the likely loss of MHC Class presentation on these cancers cells and contributing significantly to the lack of recognition by the immune system, and general tumour microenvironment immunosuppression.

Epigenetic changes in EBV+ NPC have been found to be global hypermethylation of crucial TSGs leading to downregulation of function, and this is driven by LMP1. Analyses of Histone modifications show that the level of trimethylation of Histone 3 at Lysine 27 (H3K27me3) is significantly higher in all cases of EBV+ NPC studied so far compared to controls and correlated with tumour metastasis, T3-T4 stages, chemoradiotherapy-resistance, and decreased survival [44, 45]. Furthermore, this may suggest that PRC2 with its catalytic subunit EZH2 has oncogenic functions since ATRX is a chromatin re-modeller helping PRC2 find its targets [46].

## HPV+/− Oropharyngeal SCCs

### Epidemiology

The discovery of the strong causal association of HPV to cervical carcinoma [18] by Harald zur Hausen in 1983 and the subsequent linkage to differing frequencies of other ano-genital cancers over the next several decades demonstrated the importance of this oncogenic virus. Approximately 5% of total worldwide cancers are directly linked to this virus [1–3, 19]. The link with head and neck cancers is more recent, and required much patient work from multiple groups to establish definitively [47–49]

The link between head and neck cancers and HPV was initially observed by Kari and Stina Syrjänen in 1983 when they noticed that 40% of 40 oral squamous carcinomas contained morphological and immunohistochemical evidence of HPV-infected cells within the tumours [50]. It is now clear that oropharyngeal cancers have the highest frequency of HPV oncogenesis amongst all the sites of head and neck cancers. This may suggest a peculiar susceptibility of the progenitor cell of origin to HPV here or that the viral load is particularly high at these areas after initial contact with HPV, or both. The mode of transmission is predominantly human to human via intimate contact [51].

Classical HPV+ oropharyngeal cancers arise from the epithelial crypts found in the tonsillar tissue of Waldeyer’s ring (palatine and lingual tonsils). The fact that EBV+ nasopharyngeal cancers and HPV+ oropharyngeal cancers are so close in their anatomical sites is astonishing, but also suggests that the infection of different cancer stem cells/progenitor cells of origin (cells from tonsillar crypt epithelium vs nasopharyngeal mucosal/non-keratinising squamous epithelium) plus 2 entirely different viral oncogenic drivers lead to these starkly different cancers.

Tobacco smoke is a strong oncogenic driver found to be relevant in both cancers, and it is likely that alcohol consumption synergises with tobacco smoke since it allows easier and deeper penetration of tissue surfaces due to its solvent properties [52]. Furthermore, alcohol is metabolised to acetaldehyde, and this damages DNA with adduct formation [53].

HPVs’ oncogenic properties have been studied extensively since the causal effect of infection was discovered in 1983 [18]. The same high risk cancer subtypes that cause cervical and anogenital cancers are involved, although at different frequencies. HPV 16 is highly dominant is causing Oropharyngeal carcinoma, although positive associations for HPV types 18, 33, 33, and 52 have been found at much lower frequencies [54]. This type of restricted spread of high risk HPV types is similar to the non-cervical sites in anogenital cancers.

### Viral Oncology

HPV infection of the putative cancer stem cell (CSC) in the oropharynx is likely to be an early event, and the development of invasive carcinoma likely to take 10–30 years, analogous to the cervical cancer model. In HNSCC cancers, the HPV genome has been found to be mostly integrated into the cellular genome (TCGA 2015), although episomal oncogenesis has been documented [55–57]. The high-risk HPV types include the most dominant, HPV 16, but also HPV 18, 31, 32, 33, and 52, which are the next most frequent. Over 50 subtypes have been designated high-risk types by dint of their ability to cause cervical cancers.

As seen in Fig. 2, HPV genome is only approximately 8 kb (> 21× smaller than the EBV genome). It contains 9–10 genes, most commonly E1–7 (seven early genes involved in replication and transcription of the viral genome) and two late genes (L1–L2) which encode the viral capsid proteins. L1 is most well known as the protein used for all the available and highly successful HPV (prophylaxis against infection) vaccines (bivalent, quadrivalent, and nonavalent vaccines).

#### Is there a precancerous or dysplastic phase for HPV+ oropharyngeal cancer?

There are several strands of evidence that suggest that this must be in the affirmative. First, the successful *cervical screening programmes* seen in industrialised countries have already saved hundreds of thousands to millions of women over the last 30+ years. Second, there is no evidence to suggest that the life cycle of HPV 16 and other subtypes differ drastically between anatomical sites of infection. Third, the fact that ano-genital cancers have all been proven to have the classic intraepithelial patterns analogous to cervical intraepithelial neoplasia: CIN 1, CIN2, CIN3(carcinoma in situ), anal intraepithelial neoplasia (AIN1-3), penile (PIN1-3), vulvar (Vulvar IN1-3), and vaginal (vaginal IN1-3).

These lines of evidence (circumstantial though they are) suggest that the precancerous-dysplastic phase should exist in the oropharynx as well, and that HPV+ HNSCC is analogous to other sites on the human body. This likelihood has not been acknowledged but instead dismissed in recent major reviews [4, 58•].

Masterson et al. reported on laser microdissection of dysplastic tissue surrounding the actual tumour body in 27 HPV+ HNSCC in 2016 [59••]. This paper provided strong evidence that HPV+ oropharyngeal cancers *do indeed have a precancerous phase*, although these areas are not as large compared to the HPV− oropharyngeal cancers, since the HPV-infected reticular epithelial crypts are located only within the tonsillar tissue found in the palatine and lingual tonsils, and a rapidly growing monoclonal cancer arising from these small areas will quickly obliterate surrounding tissue.

We found that there are several genes with high CNAs (copy number alterations/mutations) in the dysplastic precancerous tissue; i.e. *CDKN2A* (encodes for p16^INK4A^ and therefore expected with HPV infection) and *SYCP2* (a putative oncogene usually found in testicular tissue) are both upregulated, and *SFRP1*, *DLG2*, *CRNN*, and *CRCT1* (all downregulated). These putative dysregulated genes are strong candidates for further research into their potential suitability for early cancer or dysplastic tissue detection.

It is interesting to note that detection of HPV E6 and E7 DNA has been used successfully in terms of screening for HPV+ tumour growth recurrence as part of the primary treatment and surveillance programs [60•, 61] demonstrating the potential of this approach.

### HPV E6 and E7 Oncogenes

The most well-known oncogenes from high-risk subtypes of HPV are undoubtedly its E6 and E7 oncogenes. E6 binds strongly and avidly to p53, forming a complex with an ubiquitylation protein E6-AP, and this downregulates its tumour suppressor functions by ubiquitylation and proteasomal degradation. E7 protein on the other hand binds avidly to Retinoblastoma-associated proteins 1 and 2 (RB1/RBL1/p107 and RB2/RBL2/p130), which drives proteasomal destruction of RB and release of the E2F family of transcription factors. These E2F proteins push the cell cycle beyond G1-S checkpoint into S phase. E7 dysregulation of RB function leads to positive feedback upregulation of p16^INK4A^. This increased p16^INK4A^ expression can detected easily by immunohistochemistry, and is commonly used as a surrogate marker of high-risk HPV subtype infection. There are numerous other functions [62, 63] that have been attributed to these 2 oncogenes, but they lie out with the scope of this review.

### E2 and E5 Functions

E5 has been investigated extensively over the last 20 years, and has been shown to be a weakly transforming protein in vitro. It works best in conjunction with the other E proteins, but in sum, has been shown to induce MHC Class I downregulation (thus contributing to escape from immune surveillance and survival of the infected cell), contribute to inhibition of apoptosis, increasing cell proliferation and angiogenesis mainly via activation of EGF-R, and the downstream Ras-Raf-MAP kinase and PI3K-Akt pathways. Detailed discussion of the delineated pathways is outside the scope of this review and can be accessed through the references given [62, 64].

E2 proteins are the main transcriptional regulators for papillomaviruses and also have been shown to be important in oncogenesis. Expression of E2 strongly inhibits growth of HPV positive cells in vitro, and its main mode of action is well known: E2 represses the early viral promoter, thus down-regulating E6 and E7 expression [65].

### Genomic Mutations in Oropharyngeal Cancers

The Cancer Genome Atlas study in 2015 detailed the analyses of 279 HNSCC consisting of 243 HPV− and 36 HPV+ tumours. The study revealed that there was a high degree of genomic instability with an average of 141 CNAs and 62 chromosomal abnormalities (deletions-additions-fusions, etc).

The most frequently mutated genes were *TP53* (72%) and *CDKN2A* (22%). These are usually loss-of-function mutations and frequently occur in HPV− tumours as the actions of HPV E6 and E7 binding to these 2 genes abrogates the need for loss-of-function in these important genes for early transformative progression. *PIK3CA*, which encodes the catalytic subunit of phosphoinositide 3-kinase (PI3K) appears to be the only frequently mutated oncogene in the HNSCC genome (14%). Besides the 2 main TSGs shown above (*TP53* and *CDKN2A*), other commonly mutated TSGs or likely TSGs detected by the study were *FAT1*, *NOTCH1*, *KMT2D*, *NSD1*, and *TGFBR2.*

HPV+ tumours also showed frequent loss of function of *TRAF3*, *NSD1*, *FAT1*, *NOTCH1*, and *SMAD4*, and amplification of *E2F1*, *EGFR*, *and HER2.* HPV− tumours, on the other hand, also showed mutations frequently in *NRF2* and *KEAP1* which are important regulators of oxidative stress. These last 2 mutations appear to occur exclusively in HPV− tumours. Other mutated members of the *TP53* family such as *TP63* and *TP73* occur frequently in HNSCC, more commonly in the HPV− fraction.

The major signalling pathway involved in both HPV+ and HPV− oropharyngeal cancers appears to be the PI3K-AKT-mTOR (PI3K) pathway, as opposed to the NF-B pathway seen in EBV+ NPC. Loss of function of PTEN (phosphatase and tensin homologue), which is a negative regulator of PI3K signalling occurs in ~30% of tumours. STAT3 signalling is frequently increased in tumours although the gene itself rarely mutated [66–68]. Another important signalling pathway with significant contributions to oncogenesis in HNSCC would be the WNT pathway [69]. Other pathways involved in a smaller number of tumours would be the MAPK and NOTCH pathways.

### Epigenetics

HNSCC studies have shown that there is a global *hypomethylation* of DNA (as opposed to *hypermethylation* of DNA seen in EBV+ NPC, discussed previously). However, there is also hypermethylation and downregulation of expression of several critical TSGs such as CDKN2A, RARB, DCC, and MGMT occurring frequently, particularly in oral SCCs [70–72].

The large collection of mutations documented above shows us that there are multiple pathways involved in HNSCC, and that *accurate stratification and sub-classification* according to important risk factors such as smoking/never smoking, alcohol/never drinker, HPV+/−, betel nut, arecha nut etc. are really important in enabling the detection of the triggering and activation of these particular pathways. We shall review some of the recent findings in a couple of important recently discovered groups of these patients below:-

#### HPV+ HNSCC Patients with Heavy Smoking (> 10 Pack Years) and/or Heavy Tobacco/Betel Leaf/Areca Nut Chewing

It is eminently logical that 2 or more important risk factors with strong oncogenic drive would occur in certain groups of patients and that those cancers would have different oncogenic pathways compared to groups with just one or other risk factors. Larger epidemiological studies gave credence to this hypothesis with detection of an additive effect [37, 73, 74]. Genomic analyses using WES from India demonstrated that the mutational burden amongst HPV+ HNSCC (mostly oral SCCs) with the added mutational oncogenic drive of betel nut and tobacco chewing did not show significant mutational burden compared to the larger group of HPV− HNSCC [75].

#### HPV-Oropharyngeal Cancers with Very Few- or Silent (Copy Number Alterations — CNAs) Mutations

This subgroup has demographics which are unusual. They are usually female patients, elderly (> 70 years) or very young (< 40 years) with no history of smoking or no alcohol intake. Prognosis is better than the usual HPV− head and neck cancer patient. TP53 is typically wild-type (*wtTP53*), and there is retention of chromosome 3p [76]. They also typically demonstrate activating HRAS and inactivating CASP8 mutations [77].

These recently identified subgroups demonstrate the importance of proper and accurate stratification according to known important oncogenic drivers, as it is highly likely that pathogeneses will differ.

## Diagnosis, Staging, and Screening

Definitive diagnosis involves a histological diagnosis made from a biopsy of the primary tumour or neck lump. Routine histopathology based on standard H & E (haematoxylin and eosin) staining is usually sufficient for moderately or well-differentiated samples. Undifferentiated-poorly differentiated, basaloid morphology tumours or unknown primary tumours require further interrogation with immunohistochemistry to help define an epithelial origin (or not). As previously noted in Table 1, both of these 2 virus-induced tumours tend to be less differentiated compared to other environmental carcinogens, again emphasising their distinctive molecular pathways.

The most commonly used technique (and cheapest) for assessing HPV activity within a tumour sample is by immunohistochemistry staining for p16^INK4A^. This is as per the guidelines from the American College of Pathologists, 2018 [78]. As previously noted, this detects upregulated p16^INK4A^ protein (due to the degradation of RB) and is a surrogate marker for E7 oncoprotein function. This has been shown to have a diagnostic threshold of >70%. In cases of high suspicion but negative staining, more sophisticated (and expensive) detection of E6 and E7 mRNA transcripts (gold standard) or DNA by PCR or ISH can be utilised.

Accurate staging of disease is crucial to prognostication. HPV+ status has a major impact on prognosis, and this was evidenced properly by Ang et al. (2010) [79]. Patients with HPV+ tumours were shown to have 58% reduction in risk of death, after adjusting for age, race, tumour and nodal stage (TNM staging), tobacco smoking, and treatment assignment. (Of note is that risk of death significantly increased with each additional pack-year of tobacco smoking, which is strongly suggestive of the importance of tobacco carcinogenicity for these tumours).

The American Joint Commission on Cancer (AJCC) and the Union for International Cancer Control (UICC) released the 8th Edition of the Cancer Staging Manual in 2017 [80], and 3 important improvements were added to previous guidance for staging for HNSCC. These are depth of invasion to oral cavity cancers, extra-capsular nodal extension to nodal staging in HPV− HNSCC, and novel staging codes for HPV+ HNSCC. The 8th edition still does not include tobacco usage in its prognostication, and this may be remedied in future editions. EBV+ status and other environmental factors are not included in the hazard discrimination process in the 8th edition despite the increased evidence for their inclusion. It is likely that these will included in later editions of the Cancer Staging Manual, especially baseline plasma EBV DNA viral loads. Complete staging evaluation must include full physical head and neck examination, including with naso-pharyngo-laryngoscopy if indicated, high-resolution imaging of the relevant anatomical areas by CT, PET-CT, or MRI to detect extent of disease, be it local, regional, or distant.

Screening for HPV+ and EBV+ HNSCC disease has also advanced significantly recently. In 2017, Chan et al. [81] reported from a large randomised prospective screening trial of 20,174 asymptomatic men in Hong Kong, and showed that plasma EBV DNA detection had tremendous sensitivity (97.1%) and specificity (98.6%) for identifying NPC. Moreover, the EBV+ cancer was predominantly detected at stages 1-2(71% of total cases), which was much better than in a previous historical study (20%) [82], and progression-free survival was 97% vs 70%. This singular study has shown that it is possible to perform blood-based EBV DNA screening successfully in an endemic population for NPC and detect cancers early, thus saving lives.

Balachandra et al. (2021) [83] have reported on a meta-analyses on studies screening for HPV+ HNSCC, and their conclusions were that HPV 16 E antibody and circulating HPV DNA in blood has potential for population screening (different studies have reported sensitivities from 61-95%) [84] but as yet, complete fulfilment of criteria for population-based screening has not been achieved (for a list of the criteria, we have referenced the US Preventive Services Task Force. Procedure manual.[85] Accessed 13^th^ October 2021. https://www.uspreventiveservicestaskforce.org/uspstf/procedure-manual.).

## Management

Curative intent of treatment is of paramount consideration for any life-threatening disease. The peculiarities of these viral-induced cancers and their non-virally-induced counterparts offer unique opportunities that need to be considered. Although HPV+ HNSCC have a proven reduction of risk of death by 58% [79] compared to HPV− HNSCC matched controls, the consensus on de-escalated treatment modalities (which have reduced treatment morbidities but have an equivalent cure rate to accepted best practice) is still controversial. A couple of single-arm trials have reported that radiation dosage in virus+ HNSCC can be safely reduced [86, 87], but we await the full reports of RCTs and consensus guidance. Current best practice is summarised in Table 2.

**Table 2:** Management strategies in HNSCC

	EBV+ NPC	EBV− NPC	HPV+ oropharyngeal cancer	HPV− oropharyngeal cancer
Stages T1–2	Resection or radiation (cure rates of >80% for radiotherapy, surgery cure rates in the 90s, %)	Resection or radiation (cure rates of >80% for radiotherapy, surgery cure rates in the 90s, %)	Surgery preferred for oral cavity cancers, radiation more for pharyngeal and laryngeal cancers. Minimally invasive surgical techniques including robotics and laser scalpels in combination with better reconstruction have extended the indications for surgery.	Surgery preferred for oral cavity cancers, radiation more for pharyngeal and laryngeal cancers. Minimally invasive surgical techniques including robotics and laser scalpels in combination with better reconstruction have extended the indications for surgery.
	Use of sentinel node biopsy and elective neck dissection improves cure rates. Salvage treatment after failure of single modality treatment offers high chance of cure.	Use of sentinel node biopsy and elective neck dissection improves cure rates. Salvage treatment after failure of single modality treatment offers high chance of cure.	Use of sentinel node biopsy and elective neck dissection improves cure rates. Salvage treatment after failure of single modality treatment offers high chance of cure.	Use of sentinel node biopsy and elective neck dissection improves cure rates. Salvage treatment after failure of single modality treatment offers high chance of cure.
Stage T3	Trimodality treatment (CRT post-surgery or without surgery if high nodal volume)	Trimodality treatment (CRT post-surgery or without surgery if high nodal volume)	Trimodality treatment (CRT post-surgery or without surgery if high nodal volume)	Trimodality treatment (CRT post-surgery or without surgery if high nodal volume)
≥T3	Definitive CRT – standard is single agent cisplatin. If too elderly or concurrent renal or hearing loss, then lower fractionated doses of cisplatin may be used.	Definitive CRT – standard is single agent cisplatin. If too elderly or concurrent renal or hearing loss, then lower fractionated doses of cisplatin may be used.	Definitive CRT – standard is single agent cisplatin. If too elderly or concurrent renal or hearing loss, then lower fractionated doses of cisplatin may be used.	Definitive CRT – standard is single agent cisplatin. If too elderly or concurrent renal or hearing loss, then lower fractionated doses of cisplatin may be used.
	Anti-EGFR immunotherapy (Cetuximab) responsive but risk of distant mets.	Cetuximab should be considered if high expression of EGFR found in tumour	Cetuximab substitution for cisplatin reduces survival	Cetuximab should be considered if high expression of EGFR found in tumour
Recurrent or metastatic	Consider re-irradiation alone [87]. Consider Cetuximab alone if PDL1 expression low (CPS < 1) with multidrug chemo or clinical trial. If CPS >1, then PDL1 inhibitor or clinical trial.	Consider Cetuximab alone if PDL1 expression low (CPS < 1) with multidrug chemo or clinical trial. If CPS >1, then PDL1 inhibitor or clinical trial.	Depending on first line therapy, re-irradiation, multidrug chemotherapy combination ± checkpoint inhibitor or checkpoint-inhibitor alone might be useful options	Depending on first line therapy, re-irradiation, multidrug chemotherapy combination ± checkpoint inhibitor or checkpoint-inhibitor alone might be useful options
	If tumour burden is high, consider multidrug chemo + PDL1 inhibitor or clinical trial	If tumour burden is high, consider multidrug chemo + PDL1 inhibitor or clinical trial	If tumour burden is high, consider multidrug chemo + PDL1 inhibitor or clinical trial	If tumour burden is high, consider multidrug chemo + PDL1 inhibitor or clinical trial

CPS (combined positive score) consists of counts of PDL1+ cells (cancer cells, macrophages/monocytes, and lymphocytes) over total cancer cells counted

## Immunotherapy and Precision Medicine

The 2018 Nobel Prize in Physiology or Medicine was awarded jointly to Tasuku Honjo and James Allison for their discoveries of the immunosuppressive axes of Programmed Death molecule 1 or CD279 (PD1/PDL1) and Cytotoxic T-lymphocyte Antigen 4 (CTLA-4) respectively, and shone a spotlight on these immune checkpoint inhibitors. The use of “inhibitors of these inhibitors” allow the immune system to increase its activity, and there have been well documented RCTs with spectacular increases in disease-free survival and overall survival rates, particularly in malignant melanoma, and non-small cell lung cancer (NSCLC) but also now in other rapidly replicating and highly malignant cancers.

There appear to be a subset of patients with recurrent or metastatic HNSCC with good and durable responses to PD1/PDL1 inhibition, and the US FDA approved nivolumab and pembrolizumab in 2016 for cisplatin-refractory recurrent or metastatic disease, and pembrolizumab was approved as first-line treatment of surgically unresectable or metastatic cancers in 2019 [88–91]. CPS ≥ 1% is associated with increasing likelihood of clinical response and benefit.

Anti-CTLA4 inhibition is also being actively investigated in a multitude of clinical trials of HNSCC treatment. The inhibitory pathway of CTLA4-CD28 axis is separate from the PD1/PDL1 axis, and this means that greater release of immunosuppression could theoretically be obtained from inhibition of both axes in combination. This hypothesis has been and is being tested in clinical trials at present and has already been shown to be of great benefit in metastatic melanoma disease, demonstrating excellent remission rates, and becoming the standard of care. Of note so far, is that CTLA4 inhibitors appear to have greater toxicity/side-effects compared to PD1/PDL1 inhibitors in safety trials.

Apart from the immunotherapy cited above, another therapeutic approach could be targeting the FGFR3-TACC3 gene rearrangement, which has been picked up in small numbers in EBV+ NPC [92] and HPV+ HNSCC [77, 93]. FGFR3 inhibitors have been tried in glioma [94], bladder [95], non-small cell lung cancer [96], and cervical cancer cells [97] in vitro with good results, and there have been cases reported of good responses in vivo [98–100] in safety trials.

This serves to illustrate that certain mutations found in solid tumours are targetable with small molecule inhibitors, and these types of treatments need to be taken to full-scale efficacy RCTs (phase 3) after appropriate safety trials have been completed, and also used in conjunction with other approved therapies, as part of the armoury of weaponry of precision medicine against solid tumours.

## Conclusions

Viral and molecular oncology of these 2 HNSCCs, which are very different but yet similar in so many respects, has yielded a rich trove of knowledge and information. The viral aetiology of HNSCCs represents distinct biological pathways towards malignancy. However, more stringent stratification of patient phenotypes according to other carcinogen risk is also revealing new distinct subgroups and potentially mixed oncogenic pathways. The demonstration of pre-invasive phases (dysplasia) for HPV+ oropharyngeal cancer, analogous to HPV+ cancers at other anatomical sites, should mean that targeted screening tests for these cancers are feasible. Diagnoses of dysplastic stages and early stage cancers will reduce mortality. Better staging, prognostication, and management will help improve patient outcomes, especially with the advent of immunotherapies and precision medicine.
